# Three in a bed and changing allogeneic transplant outcomes with a second, non-engrafting cell source

**DOI:** 10.3389/fimmu.2026.1904787

**Published:** 2026-09-21

**Authors:** Nathan Jeffreys, Kay Poulton, Robert Wynn

**Affiliations:** 1 Pediatric Blood and Marrow Transplant Programme, Royal Manchester Children’s Hospital, Manchester, United Kingdom; 2 Transplantation Laboratory, Manchester University NHS Foundation Trust, Manchester, United Kingdom

**Keywords:** graft-vs-leukemia effect, hematopoietic cell transplant, leukemia, leukocyte, non-engrafting cellular therapy, T-replete

## Abstract

Relapse remains the most significant cause of mortality after allogeneic transplant for leukemia despite advances in understanding of disease biology and newer, targeted treatment modalities. Cure after allogeneic transplant is mediated through the graft-vs-leukemia effect, as donor immune cells recognize and reject leukemic cells, largely through recognition of mismatched HLA and/or minor histocompatibility antigens. There is growing evidence that adjuvant but non-engrafting cellular therapies, either alone or in conjunction with conventional allogeneic transplant, improves leukemia outcomes and immune responses to such therapies have also been described during solid-organ transplants and following double cord unit transplantation, and are there associated with clinical consequences. We review these immune responses induced by third party and non-engrafting leukocytes and suggest possible mechanisms for the action of such cells. We further suggest that improved understanding of the nature of such responses might allow development of transplant technologies to make allogeneic transplants both better and safer, by targeting the immune response of the allogeneic immune system more specifically to leukemia.

## Introduction

### Relapse remains a significant barrier to improved outcomes in leukemia

Outcomes of patients with leukemia have improved substantially over recent decades as a direct result of advances in our understanding of the underlying tumor biology and its molecular and genetic features. The use of new targeted treatments, alongside existing treatment options and advances in supportive care, has led to improvements in overall survival and significant reductions in treatment-related toxicity. Hematopoietic stem cell transplantation is part of the therapeutic landscape and is required often for cure of disease but the commonest cause of treatment failure, even with such transplant, remains disease relapse. There remains therefore significant unmet need to improve outcomes for children and for adults with difficult-to-cure leukemia.

### How transplant cures leukemia

Hematopoietic stem cell transplantation (HSCT) has become a mainstay of treatment for hematologic malignancies and cures many patients who are refractory to other treatment modalities, or who have relapsed after previously achieving remission. In general, HSCT might result in cure through dose escalation of chemotherapy or radiotherapy given during conditioning since such escalation might overcome resistance, and this is the principle of autologous transplantation. However, the principal benefit in allogeneic transplant is immunologic, and through a graft-versus-leukemia (GVL) effect during which donor-derived immune cells recognize and destroy host leukemic stem cells and their progeny.

Transplant remains the oldest and numerically most successful immune therapy of malignancy. In recent years other immune therapies of cancer have reached the clinic, including monoclonal antibodies, CAR-T approaches, co-stimulation blockade that blocks an inhibitory signal to tumor-reactive T-cells, tumor infiltrating lymphocytes (TIL), TCR-T (engineered T-cells with a transgenic T cell receptor that recognizes tumor antigen expressed with HLA) and Bispecific T-cell engager (BiTE) antibody therapies that brings T cells to the tumor with an antibody that binds both tumor and T cells (all approaches summarized in **Table 1**). The plethora and efficacy of such treatments further indicate that cancer can be controlled by immunologic means and that this is true for both hematologic and solid tumors.

**Table 1:** Summary of key immunotherapy modalities.

Immunotherapy modality	Mechanism of action	Licensed examples
Cell therapy based		
Allogeneic HCT (1)	Primarily mediated by graft-vs-leukaemia effect	N/A
Chimeric antigen receptor (CAR-T) (2)	Genetically engineered autologous T cells that possess an artificial transmembrane protein that binds to tumour surface antigens and then initiates intracellular signalling, to promote antitumour T cell mediated cytotoxicity	Tisangenlecleucel
T-cell receptor engineered T-cell therapy (TCT-T) (3)	Genetically engineered autologous T cells that possess a modified T cell receptor that binds to tumour intracellular proteins presented on HLA molecules, to promote antitumour T cell mediated cytotoxicity	Afamitresgene autoleucel
Tumour infiltrating lymphocytes (TILs) (4)	Autologous T cells from within host tumour are isolated and expanded ex vivo before reinfusion, promoting antitumour T cell mediated cytotoxicity	Lifileucel
Drug- or antibody- based		
Monoclonal antibodies and antibody-drug conjugates (5)	Range of mechanisms of action, including: • Antibody dependent cellular cytotoxicity • Antibody dependent phagocytosis • Compliment mediated cytotoxicity • Direct cytotoxicity and apoptosis • For ADCs, delivery of cytotoxic payload	Rituximab (anti-CD20) Brentuximab (anti-CD30) Gemtuzumab ozogamicin (anti-CD33)
Bispecific T cell engagers (BiTEs) (6)	Simultaneously binds to both T cell receptor and tumour cells, promoting direct T cell mediated cytotoxicity	Blinatumomab (CD3 x CD19)
Immune checkpoint inhibitors (7)	Blocks inhibitory immune checkpoints, e.g. PD-1, CTLA-4, restoring anti-tumour T cell mediated cytotoxicity	Pembrolizumab (PD-1) Nivolumab (PD-1) Ipilimumab (CTLA-4)
Tumour vaccines (8)	Induce tumour specific immunity by presenting tumour antigens to prime immune mediated recognition and responses	Clinical trials only

**Table 1** summarizes the main immunotherapeutic treatment modalities currently available or in development.

T-cells are the principal mediator of GVL. There is increased relapse when the graft is depleted of donor T-cells (9), or when there is a high level of immunosuppressive drug (10). Furthermore, remission can be achieved where there is post-transplant disease relapse with donor T-cell infusion (11). Donor T-cells recognize recipient leukemic cells through recognition of HLA-mismatch, or minor Histocompatibility Antigen-mismatch (MiHA), namely mismatched peptides presented by matched HLA. GVL is closely associated with Graft versus Host Disease (GVHD) since the target antigens are shared, and not leukemia-specific, and GVHD remains an important and much feared cause of morbidity and mortality after allogeneic transplant.

HLA matching between transplant and donor at the major HLA loci contributes to successful engraftment and long-term graft function in recipients of stem cell transplant. Recipients with a HLA-matched donor do better than those without such a donor (12). Relapse rates may be reduced when there is HLA-mismatch as the leukemia is more foreign to the donor, but this comes at the cost of increased incidence of GVHD.

When there is major HLA mismatch between donor and recipient, as during haplo-identical or other mismatched donor transplant, loss of HLA expression on the leukemic cells contributes to relapse after transplant and occurs through several mechanisms. This was explored by Vago et al. who has described both genomic loss of the mismatched HLA genes and epigenetic modification to downregulate mismatched HLA gene expression in the leukemic clone at relapse (13–15). At relapse after haploidentical transplant, there is copy-neutral loss of heterozygosity (CN-LOH), i.e. duplication of the compatible haplotype and loss of the mismatched haplotype. This implies that major HLA-mismatch is a GVL target and by down regulation of this mismatched antigen, there is reduced immunologic recognition of the disease by the donor which also limits the utility of therapeutic options such as donor leukocyte infusion (DLI). They have recently presented evidence that this mechanism of relapse relates to transplant type, with reduced rates of genomic loss of the mismatched HLA at relapse in CB transplant compared to MUD, likely as the mismatches are on both haplotypes and the mismatched antigen expression cannot be achieved replacement of the mismatched haplotype by the matched haplotype.

### Improved therapeutic options are still required

Despite improved outcomes with hematopoietic stem cell transplantation, relapse after transplant remains an issue and significant cause of treatment failure (16). This occurs in 20-70% cases and depends on several factors including disease subtype (17), donor factors (18), the presence of residual disease before transplant (19) and the intensity of conditioning therapy (20). Its efficacy is derived from donor T cell action, and relapse might be expected to be reduced by better focusing the recognition of host malignancy by donor T cells. Existing therapies such as DLI are used in select cases only to augment the GVL effect. More widespread use is limited by excess GVHD, leading to high treatment related morbidity and mortality (21, 22).

Substantial and long-standing evidence suggests that direct leukocyte-induced responses may have a beneficial effect in the treatment of hematological malignancies. We describe the history of such therapies that describe leukocyte-induced immune responses in allogeneic transplantation and how this may influence clinical decisions around donor source and adjuvant therapies to reduce relapse risk and improve outcomes. Studies were identified through PubMed indexing and analysis of references for selected landmark studies; this approach is non-exhaustive but permits a broad oversight of the current landscape and provides perspectives for future work in the area.

## Leukocytes and their effects

### Historical perspectives on leukocyte transfusion

The ability of blood transfusion to induce remission of acute leukemia was first reported in the 1960s, with several reports of prolongation of remission and even some examples of spontaneous remission following leukocyte-replete blood transfusion. In 1967, Schwarzenberg (23) reported on 24 patients with ‘acute leukemia in the evolving phase’ who were treated with leukocyte transfusion to treat concurrent infection, and noted six complete remissions and three incomplete responses in the absence of concomitant chemotherapy, therefore suggesting an antileukemic effect of the transfusions themselves. Subsequent studies suggest that the anti-leukemic effect of blood transfusion was reduced following leukocyte reduction, such as irradiation or white cell filtration (24), suggesting the importance of allogeneic leukocytes as the key agent in the anti-leukemic effect seen.

A more recent case report by Creasey et al. details a 45-year-old woman with poor-risk AML who received sixteen irradiated granulocyte pools on days 82–93 for supportive treatment during a period of severe neutropenia and suspected aspergillus lung infection. A sustained improvement in neutrophil, lymphocyte and platelet count was seen following the granulocyte infusions, and bone marrow evaluation on day 105 showed spontaneous morphological and cytogenetic remission with <5% blasts. The team went on to consolidate with a 10/10 HLA-matched MUD on day 130 and report sustained complete remission at 1 year follow-up.

### Leukocytes in solid-organ transplant medicine

The above examples suggest that allogeneic leukocyte transfer may have a role in leukemia treatment but do not specify the underlying mechanisms for the responses seen.

In the solid organ transplantation setting, specific HLA-mediated immune responses following (unintended) leukocyte transfer are well described and such immune responses are an important mediator of graft rejection (25). Frequent blood transfusions can provoke HLA sensitization, i.e. the development of HLA-specific immune responses. Evidence suggests that in solid organ transplantation such an immune response is strongly associated with graft rejection (**Figure 1A**). This remains evident in the post-transplant period where blood transfusion is adversely associated with graft survival, specifically if the transfusion bears the same HLA-mismatch with the patient as the transplanted kidney. Hassan et al. (26) reported on 1104 renal and simultaneous kidney-pancreas transplant recipients in whom post-transplant blood transfusion was shown to be independently associated with the development of *de novo* donor-specific HLA antibodies, and this was associated with graft loss even when the antibodies resulting from such immunological priming were not donor-directed. The mechanism underlying the increased likelihood of graft loss in transplant recipients who develop transfusion-induced HLA-specific antibodies is unclear. It is evident though, that transferred leukocytes are highly immunogenic and prime an *antigen specific* immune response by the recipient directed against any HLA-mismatch, which then has the potential to become directed against the transplanted organ leading to rejection. It is possible that similar immune responses to third party leukocytes are transferred to leukemic cells and explain the beneficial effects of leukocytes in patients with leukemia.

**Figure 1:**
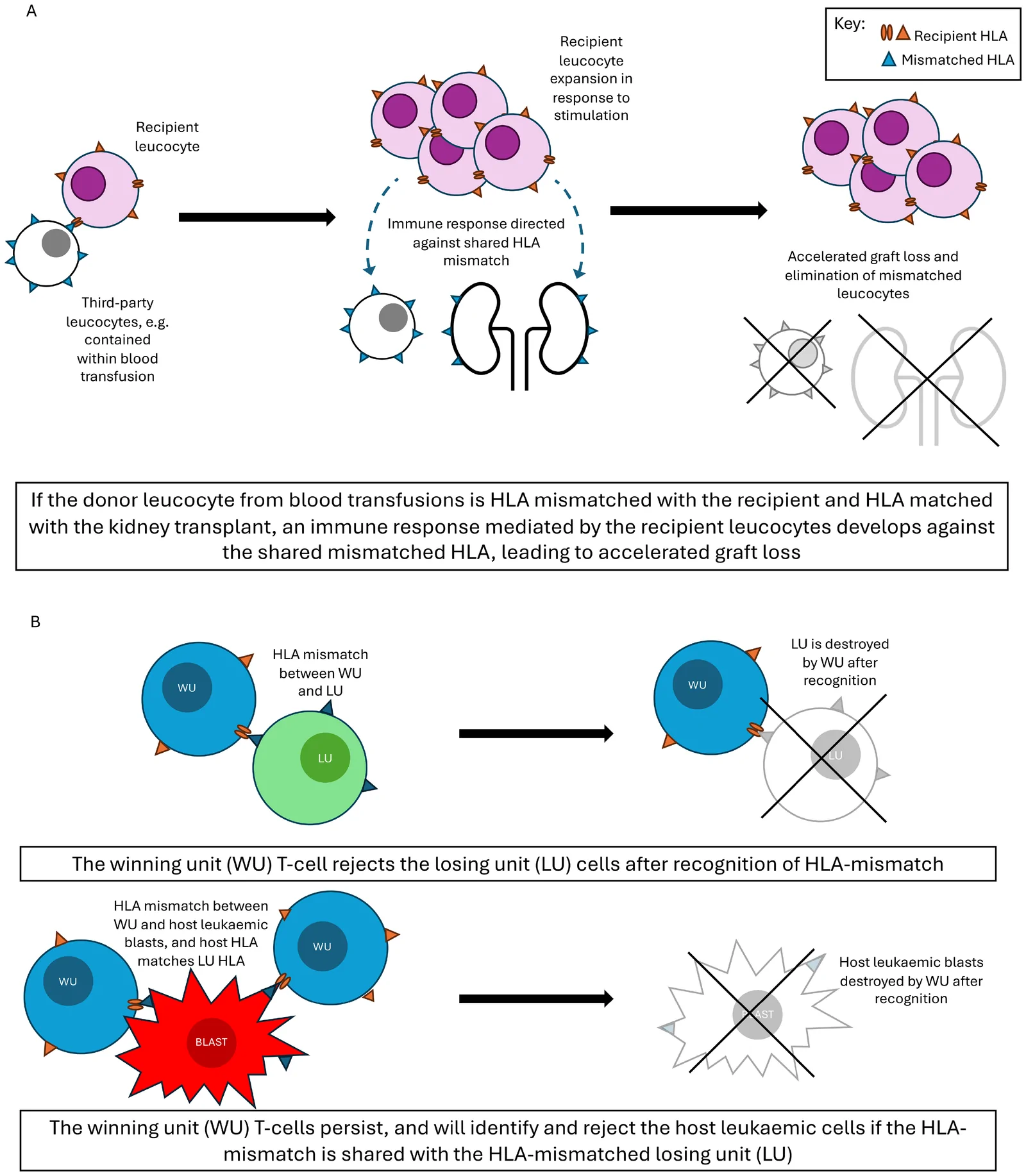
**(A)** Diagram showing that presence of mismatched HLA expression on the surface of third-party leucocytes (e.g. from blood transfusion) can be recognised and potentiates an immune response. If the transplanted solid organ, e.g. kidney, shares the same HLA mismatch with the third-party leucocyte, this can lead to both elimination of the third-party leucocytes and accelerated graft loss, as in Hassan et al. **(B)** Diagram showing the mechanism behind great anti-leukemic effect in mismatched double CBT, wherein shared mismatch between the LU and host promotes GvL effect by the WU which has been “primed” by rejection of the LU during early engraftment (26).

### T-replete cord blood as an attractive transplant source in high-risk malignancy

Cord blood (CB) transplantation has been shown in several studies to improve outcomes in high-risk malignancy compared to other cell sources. CB transplants are more often performed with HLA-mismatch and T-cell replete donors than other cell sources, which may contribute to the improved survival rates seen in several papers. This is mechanistically plausible as GVL is optimized through identification of mismatched HLA. In CB transplant, the mismatched HLA loci are spread throughout both inherited copies of the major histocompatibility complex which encodes the HLA genes and not positioned on the same strand of genes as in a haploidentical transplant, therefore genomic loss as a mechanism of HLA loss is reduced, leading to lower relapse rates. We have previously published, using UK retrospective data from all children transplanted with AML, the incidence of relapse is reduced following transplant in all children if T-cell replete cord blood (TRCB) is used as the transplant cell source. The differences are particularly marked in children with the highest risk disease, those with measurable residual disease (MRD) at the time of transplant. In those with MRD, there was a very significant difference in relapse-free survival (RFS), of 50% in the TRCB group and 21% in those transplanted with other donors. In those without MRD, RFS was improved in the TRCB group, but not significantly so.

Despite that TRCB transplants were more often mismatched and were more often performed T-cell replete, the rates of chronic GVHD were lower in this group (5% vs 19%) and the composite endpoint of chronic GVHD-free, relapse-free survival (GFRS) was much better in this TRCB cohort than in those transplanted from other cell sources (48% vs 11%). Others have reported similar findings that in those AML patients at highest risk of relapse after transplant, the use of TRCB has reduced relapse rates and better RFS and GRFS than other cell sources. Most notably, Milano et al. published institutional data from Seattle that was remarkably like our UK retrospective data. Mismatch at multiple HLA loci between donor and recipient has been associated with reduced post-transplant relapse risk, however overall survival was comparable between CB and HLA-matched unrelated donor transplantation. Some guidelines for CB selection specifically advocating for the selection of mismatched CB units to utilize HLA-mediated GVL immunogenicity and reduce relapse risk in the setting of high-risk malignancy. However, there is the potential that such benefits from lower relapse risk after CB transplantation may be offset by higher rates of non-relapse mortality in some cohorts.

### Double CB transplant setting

In double CB unit transplantation (DCBT) for hematological malignancy, where two cords are used to increase cell dose for heavier subjects, it has been shown that HLA alloimmune responses have a beneficial impact on overall survival (27). When DCBT is performed, one cord tends to predominantly engraft - termed the “winning” unit - while the other cord is actively rejected by the first cord in a HLA-mediated process - termed the “losing” unit (28).

We proposed that when there is shared HLA mismatch between the “losing” unit and the host (and therefore host-derived leukemic cells), the T-cell response, primed by the “winning” unit against the “losing” unit, is transferred and rejects the leukemic clone, clinically resulting in reduced relapse rates (**Figure 1B**). Evidence for this comes from a retrospective registry study wherein there was a significant difference in relapse risk with increasing HLA disparity only when these mismatches were shared with the recipient (RR 7% vs 29%, P<0.005), and especially if the shared mismatch was at HLA-A. Indeed, if there were 2 such shared mismatches including one at HLA-A, then the relapse rate in this large series was zero. Such shared mismatches did not increase the risk of Graft versus Host Disease (GVHD) or Transplant-related mortality (TRM), unless the shared mismatch was at class II, including HLA-DR. The results are dramatic, and there is no increased GVHD or TRM where there is such shared mismatch. Further prospective studies are needed to elucidate the mechanisms around beneficial immune responses in DCBT further.

### Leukocytes for treatment of high-risk leukemia

Allogeneic leukocyte transfer can therefore induce antigen-specific T-cell responses in the solid organ setting, and during DCBT. There is emerging evidence that the direct transfer of allogeneic leukocytes may prime an immune response against leukemia cells to promote disease control. A range of potential leukocyte sources have been reported in the literature, and there is varying nomenclature with the terms “non-engrafting alloreactive cellular therapy (NEACT)”, “non-engraftment cellular therapy”, “microchimerism” and “microtransplantation” variously used in both the pediatric and adult setting. We propose that many previously disparate observations may be united by this mechanism, resulting from the transfer of donor leukocytes that do not engraft which have prompted an immune response responsible for the improvement in outcomes seen, and will use the term NEACT throughout. **Table 2** summarizes studies reporting on the use of NEACT in hematologic malignancy.

**Table 2:** Summary of published studies using different modalities of (Non-Engrafting) leukocyte transfusion for treatment of AML.

Author/ reference	Study design	Disease stage	Number of patients	Demographics	Comparator	Donor	Outcome	Comment
Leukocyte/granulocyte products								
Schwarzenberg (1967) (23)	Case series	Various	24	Adults Age not specified	None	Chronic myeloid leukaemia patients	CR in 5/21, partial response in 4/21, non-response in 12/21.	First identified a link between “secondary syndrome” (fever, rash) with efficacy of anti-leukemic effect. Limited by lack of comparator.
McCredie (1878) (29)	Cohort study	Early	38 (26/38 AML+ 12/38 ALL)	Adults 15 – 76 yrs	Age-matched controls	Chronic granulocytic leukaemia patients	CR rate at end of induction 76% vs 69% in control group (n.s.)	In patients < 50y, median survival improved form 70 weeks to 115 weeks. Limited by lack of detail on cohort assignment and study design.
Freireich (2013) (30)	Randomised control trial	Early	108	Adults 19 – 84 yrs	Irradiated vs non-irradiated granulocytes	Healthy relatives and volunteers, single donor units (non-HLA typed)	Median survival 4.1 months in irradiated vs 2.2 months in non-irradiated (n.s.)	G-CSF mobilised cells. Non-inferiority of non-irradiated granulocyte units without excess GvHD.
Creasey (2016) (31)	Case study	Late/ Refractory	1	Adult 45 yrs	None	Irradiated pooled granulocytes	Spontaneous remission. Alive at 12-month follow up	Received RIC 10/10 HLA MUD transplant as consolidation. Limited as n=1 report.
Hiwarker (2020) (32)	Case series	Relapsed/ Refractory	5	Children 1y – 7 yrs	None	Irradiated pooled granulocytes	4/5 in remission at latest follow up of 14 – 40 months	Granulocytes given for treatment of concurrent infection. Limited by lack of comparator.
Borrill (2023) (33)	Single arm prospective trial	Relapsed/ Refractory	10	Children 2 – 14 yrs	None	Irradiated pooled granulocytes	9/10 achieved haematological remission; 5/10 remain alive and in remission with 12.7 months median follow-up	7 doses of granulocytes given without any transfusion reaction. Limited by lack of comparator (did use historical cohort for some comparisons, e.g. engraftment kinetics, but not all outcomes).
Milano (2026) (34)	Single arm prospective trial	Refractory	28	Adults and children 10 – 63 yrs	None	Dilanubicel (expanded pooled CD34+ product)	96.4% survival at median follow-up of 458 days	No grade III/IV aGvHD or cGVHD reported. Limited by lack of comparator (did use historical cohort for some comparisons).
Haploidentical peripheral blood stem cells (PBSCs, containing CD34+ and other myeloid and lymphoid cells) products								
Medina (2008) (35)	Single arm prospective trial	Late/ Refractory	10 (8 AML)	Adults 27 – 83 yrs	None	Partially HLA-matched (>4/10) relatives	2/10 had clinical response (1 unintentional engraftment, subsequently died of GvHD). Relapse on day +179.	G-CSF mobilised cell product. Limited by lack of comparator.
Colvin (2009) (36)	Single arm prospective trial	Late/ Refractory	13	Adults 16 – 82 yrs	None	HLA-haploidentical donors	CR in 3/13 (23%) and partial response in 7/13 (54%)	G-CSF mobilised cell product. Limited by lack of comparator.
Guo (2011) (37)	Randomised control trial	Early	30	Adults 60 – 88 yrs	28 controls	Relatives (21/30 had 5/10 mismatch, 9/30 had 4/10 mismatch)	Two-year DFS 38.9% vs 10.0%. Two-year OS 39.3% vs 10.3%.	G-CSF mobilised cell product (3 infusions).
Guo (2012) (38)	Single arm prospective trial	Early (in CR1)	101	Adults and children 9 – 65 yrs	None	Relatives (32/101 had 4/10 mismatch, 61/101 had 5/10 mismatch, 6/101 had 6/10 mismatch, 2/101 had 8/10 mismatch)	6-year OS rates 89.5% (low risk) and 65.2% (intermediate risk) 6-year LFS rates 84.4% (low risk) and 59.2% (intermediate-risk)	G-CSF mobilised cell product. 3 infusions. Limited by lack of comparator.
Forès (2014) (39)	Case series	Early	3 (+1)	Adults 64 – 72 yrs	None	haploidentical 4/8 mismatch (also reported on a single 7/8 mismatch)	All alive and in CR at latest follow up of 7 – 11 months (7/8 mismatch patient had early relapse but alive at 12 month follow up)	G-CSF mobilised cell product. Single infusion. Limited cohort size and lack of comparator.
Yuan (2014) (40)	Single arm prospective trial	Early	1	Adult 64 yr	None	Haploidentical relative (6/12)	No improvement. Died of disease progression 4 months post-discharge	G-CSF mobilised cell product. Single infusion. Limited by n=1 cohort.
Jing (2017) (41)	Single arm prospective trial	Early	29	Adults 57-77 yrs	None	Relatives	1-year DFS 47.3%. 2-year DFS 36.9%. 1-year OS 72.2%. 2-year OS 59.6%.	G-CSF mobilised cell product. Limited by lack of comparator.

**Table 2** summarizes the existing literature on non-engrafting cellular therapies.

#### Granulocytes

The optimal donor leukocyte source to achieve this effect remains unclear. We have previously reported (32) on our observation that pooled buffy coat granulocyte infusions can provoke T-cell expansion following cord blood transplantation for relapsed and refractory pediatric leukemia (see **Figure 2**). 5 patients received ten to twelve irradiated pooled granulocyte infusions for concurrent infection during the peri-transplant period. In 4 of 5 patients, very early CD8+ biased T-cell expansion was found, which preceded myeloid engraftment. A durable remission was invoked in 4 of 5 patients (80%) after a median of 26 months post CB transplant. The remaining patient died as a result of veno-occlusive disease and multiorgan failure related to conditioning.

**Figure 2:**
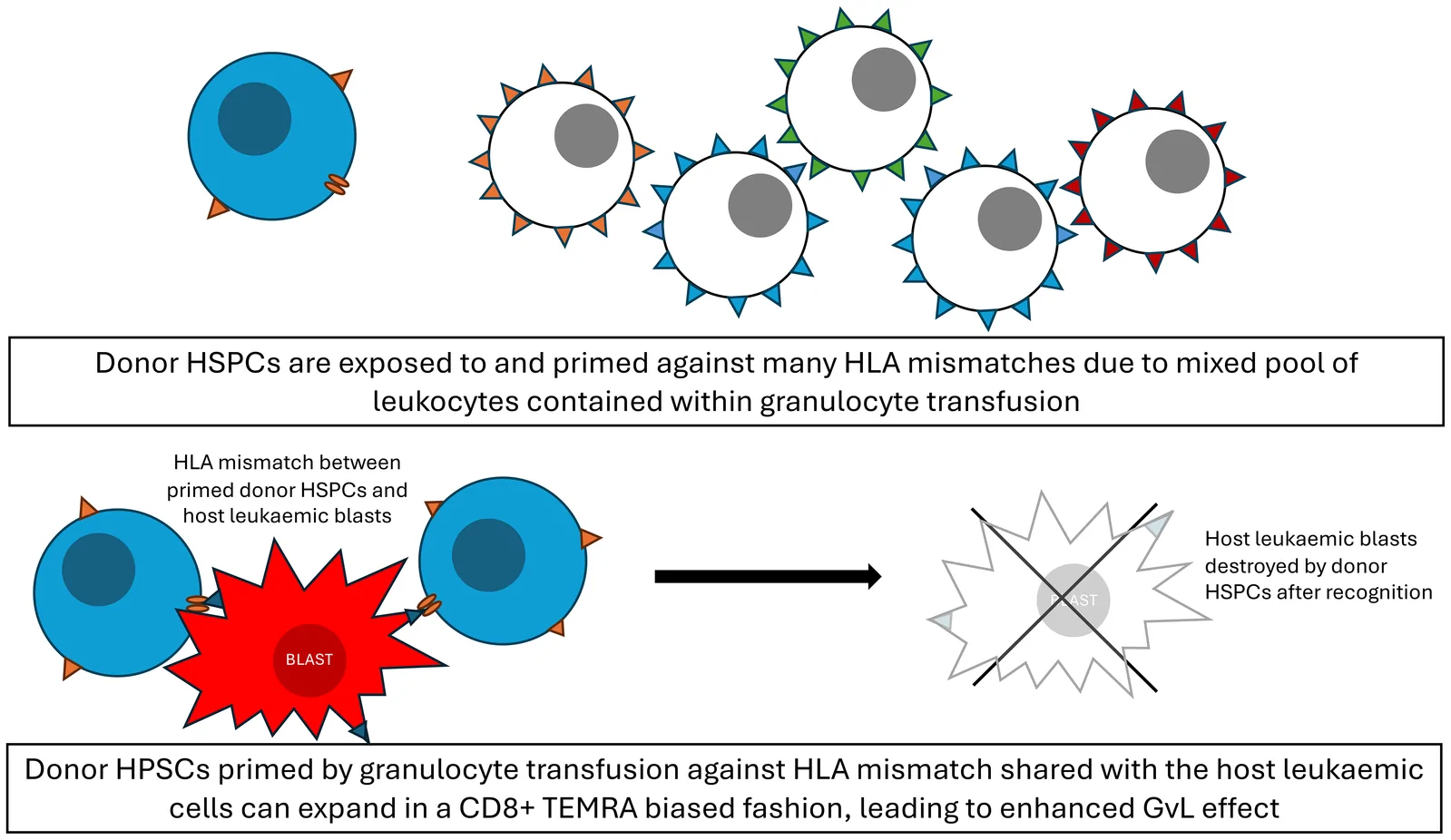
Diagram showing the mechanism behind granulocyte driven immune responses. When donor HSPCs are exposed to and primed against HLA mismatch, and if there is shared mismatch between the granulocyte and the host leukaemic cell, there is CD8+ expansion leading to enhanced GvL effect.

Following this observation, a prospective, single-center, phase I/II clinical trial (the GRANS trial, registered trial ID: NCT05425043) was opened to further evaluate the role of granulocyte transfusion in very high risk acute pediatric leukemia following relapse after previous transplant. We have previously described improved outcomes using this approach, with clinical CRS and early reproducible leukocyte expansion, which is mainly CD8+ and TEMRA biased, with improved overall survival. Importantly this approach did not lead to excess GVHD and was largely well tolerated. The phenotype switch from naïve T cells in the infused CB unit to memory T cells in the expansion suggests that this is an antigen-driven expansion, and these antigens are presented by the granulocytes and antigen-presenting cells (monocytes and B cells) within this blood product.

Our use of pooled buffy coat granulocytes is an attractive option within the UK as they can be provided as a “ready-made” product by NHS Blood and Transfusion Service, and so avoid the complexities associated with directed apheresis for leukocyte donation. Each unit of granulocytes given within the UK is a pooled product which contains the cellular component of the buffy coat extracted from 10 whole blood donations from separate donors. In addition, UK granulocyte products are relatively lymphocyte- and monocyte-rich when compared to apheresis granulocyte products used in other countries. The use of multiple donors provides the potential for multiple loci of HLA mismatch and may also provide additional mechanisms of immunogenic effect such as a greater number of minor histocompatibility antigen mismatches to promote additional NK-cell mediated alloreactivity.

Alternative third-party products have been explored in place of pooled granulocyte transfusion to augment the GVL response. Milano et al. report on 15 patients with acute leukemia treated with a CD34+ product derived from six to eight HLA-untyped umbilical cord blood units, with *in-vitro* expansion using the Notch ligand pathway, and show that this product, DVX101, can augment the anti-leukemic effect of “traditional” HLA-mismatched cord blood transplants. This approach is beneficial because it utilizes an off-the-shelf product rather than resource intensive pooled granulocyte products, allowing streamlining and standardization of delivery. Laboratory evaluation showed evidence of early myelomonocytic recovery derived from DVX101 that was no longer present by day 14, and early transient lymphocyte expansion derived from the unmanipulated CB unit which preceded neutrophil recovery in all patients. Importantly, following this protocol, the authors report remission rates of 100% at a median follow-up of 12 months, with comparable rates of GVHD with historical controls. No donor-recipient matching to the units contained within the DVX101 product was performed, although the authors hypothesize that HLA presentation by 3rd party donor leukocytes and subsequent T cell activation is likely a key mechanism for the anti-leukemic effect observed. The reported T cell expansion is similar to that described following granulocyte infusion, with mostly cytotoxic CD8 T cells, expressing granzyme and perforin. There was similar fever and CRS-like response as in our study, and there were similarly low rates of GVHD and leukemic relapse reported. Because of the T cell phenotype, it is possible that the third-party leukocytes, it is likely there are antigen-specific immune responses and that these may underpin a clinical benefit in reducing relapse without increasing GVHD.

#### Haploidentical peripheral blood stem cells

Haploidentical peripheral blood stem cells have been used in multiple trials as a leukocyte source for inducing a response to improve outcomes. Guo et al. reported results of a trial of elderly patients with AML, where patients were randomized to receive or not mobilized haploidentical peripheral blood stem cells (PBSC, containing CD34 positive and other myeloid and lymphoid cells) during induction and consolidation chemotherapy. Those who received PBSC had a higher RFS at 2 years (40% vs 10%).

Reagan et al. (42) treated 41 patients with refractory, relapsed malignancy with haploidentical CD34+ cells and escalating T-cells, after low dose TBI. Durable disease responses were obtained in 14 of 26 patients with hematological malignancy, 9 of which were described as major. Interestingly there was a cytokine release type syndrome in the patients, with rash and fever and leukocytosis, and there was elevation of certain cytokines including IFNγ and IL-6. There was no engraftment of the haploidentical donor cells in either the Guo or the Reagan study suggesting the response is a distinct mechanism compared to donor-lymphocyte infusions used in the post-transplant period to improve engraftment responses.

The combination of cord blood transplant with progenitor cells from partially HLA-matched adult donors (haplo-cord transplant) has been used in Europe and the USA over the past two decades. The adult donor graft is CD34 selected and provides early hematopoiesis, but durable engraftment derives from the cord blood graft (CD34 selected haplo-cord). Neutrophil recovery is prompt and rates of acute and chronic GVHD are low. Where myeloid and, particularly, T cell engraftment is derived from the cord blood donor, then relapse rates are reduced compared to when they are derived from the haploidentical donor.

Recent Chinese studies combine cord blood grafts with T-replete haplo-identical grafts (unmodified haplo-cord). The haplo-identical graft usually establishes dominance and prolonged UCB chimerism is rarely detected. Comparison studies suggest considerably decreased rates of relapse and improved outcomes, compared with either haplo-identical transplant or CB transplant, particularly in patients with advanced leukemia. Interestingly, using different graft manipulation may preferentially promote CB engraftment. Most reported studies are institutional, and the variable backbone and engrafting cells make exact interpretation, including interpretation of mechanism difficult. A recent review of these various studies includes a meta-analysis of six studies and confirmed an association of haplo-cord with improved OS (Hazard ratio (HR) 0.68, p < 0.0009), PFS (HR 0.67, p = 0.009) and time to relapse (HR 0.435, p < 0.0001).

Two recently reported Chinese trials both utilizing the combination of unmanipulated haploidentical cells and allogeneic transplant, including CB transplant. In both reports, the haploidentical cells that engraft, and the CB or other donor acts as the donor leukocyte source. Yu et al. compared the efficacy of haploidentical PBSCs plus unrelated cord blood with that of haploidentical PBSCs plus bone marrow as transplantations for patients with hematological malignancies. Transplantation of haploidentical PBSCs plus unrelated cord blood resulted in superior disease- free survival and overall survival in patients with hematological malignancies, with a more satisfactory safety profile.

Zhou et al. build on previous experience using co-infusing haplo-identical HSC at the same time as CB transplant, usually used to hasten engraftment (from the haplo-CD34 cells, whilst preserving the GVL from the T-cell replete CB unit). They report on a multicenter, randomized, phase 3 trial wherein eligible adult patients aged 18–60 years with available haploidentical donors and suitable for allotransplantation, were randomly allocated (1:1) to receive haplo-cord HCT (n = 134) or haplo-HCT (n = 134). The 3-year overall survival (OS) was the primary endpoint in this study and was significantly better in the Haplo-Cord group. Most engraftment was from the haplo-identical donor, although transient microchimerism was detected from the CB unit in a number of cases. Again, we posit that improved outcomes are a result of the effect of non-engrafting donor leukocyte transfer.

### Possible mechanisms of anti-leukemic effects

There are several potential distinct mechanistic explanations for improved outcomes after NEACT (summarized in **Table 3**). Evidence in this field of non-engrafting cellular therapy has been well summarized by Krakow and colleagues (43). In many reports, the usage of NEACT cellular therapy induces an inflammatory immune response, and this may have direct effects on clinical outcomes. This inflammatory response manifests clinically as cytokine release syndrome, which is associated with elevated IFNɣ, and this has been described to upregulate HLA expression on leukemic blasts, making them more visible to the engrafting donor-derived immune system.

**Table 3:** Possible mechanisms of anti-leukemic effects in non-engrafting cellular therapies.

Possible mechanisms of anti-leukemic effects in non-engrafting cellular therapies	
Inflammatory response	Bystander anti-leukemic effect of cytokine storm during rejection of non-engrafting cells by graft or host Also, IFN-Y production can upregulate HLA class II expression on leukemic blasts, enhancing recognition and elimination Evidence for: cytokine release syndrome-type clinical features
Direct alloreactivity and anti-leukemic action of active immune cells within NEACT	HLA and MiHA mediated alloreactivity against tumour by non-engrafting cells Evidence for: reports of antigen specific T cell responses in NEACT setting
NEACT primes immune response of transplant against leukaemia	HLA and MiHA mediated rejection of NEACT by graft primes a response against shared mismatch in tumour Evidence for: analogous to rejection described in DCBT setting, solid organ setting after blood transfusion, etc.

**Table 3** summarizes the possible mechanisms that have been proposed for the anti-leukaemic effects seen in non-engrafting cellular therapy trials.

Secondly, it might be that the active immune cells present in non-engrafting cell products have a direct anti-leukemic effect through HLA-mediated recognition and action. It is well described that the presence of detectable, specific T cell responses correlate with improved survival after HSCT (44). In work by Guo et al. using HLA-mismatched PBMC, the presence of detectable anti-WT1 CD8+ T cell responses were detectable in the majority of patients, and was correlated with superior survival outcomes compared to those who did not develop such responses (38).

A third explanation for efficacy of NEACT in combination with transplant is that the NEACT might prime an immune response of the transplant at shared mismatch. This is analogous to the HLA mediated renal rejection described above where the transfer of allogeneic leukocytes that share mismatch between the renal transplant and the patient and is also like evidence from double cord transplant wherein the losing unit can induce a specific immune response at shared mismatch between the LU and the patient.

Other mechanisms are likely to be relevant to the complex interplay of host, graft and NEACT and therefore will influence the outcomes seen. IFNɣ and other cytokines are likely to influence alloimmune responses and leukemic cells in a mechanism distinct to the HLA-mediated responses described above. Many studies have utilized mobilized PBSC which have distinct biological features compared to other stem cell sources. Further biological studies are required to explore key drivers of anti-leukemic effects, however given the above studies the authors feel HLA-mediated mechanisms are likely significant and are attractive as such responses can readily optimized by selecting specific mismatch within both graft and NEACT components.

### Possible limitations and safety considerations

The above approach of utilizing non-engrafting therapies alongside transplant may provide beneficial outcomes but also has important safety considerations and may come with higher toxicity. The use of deliberately mismatched components increases the risks of immunological mediated reactions in the peri-transplant period, with several papers reporting increased rates of clinically relevant cytokine-mediated responses. Such responses are frequently manageable in experienced centers; for example, in our own trial utilizing granulocyte transfusion to augment CB transplantation in the pediatric population we had substantial rates of grade 1–2 CRS however minimal excess PICU admissions (33), and such findings are reported by others (34). GVHD rates may be theoretically higher due to increased HLA mismatch, however several of the above studies state that no excess GVHD was noted, although this may be limited by limited follow-up periods and small cohort size. Other post-transplant immunological complications such as alloimmunization and graft failure are not yet well characterized but may occur at higher rates.

Patient selection is likely to be important; high-risk leukemia patients with good performance status stand to benefit the most and tolerate higher rates of NEACT treatment-related toxicity better, but in more frail populations or areas where conventional transplantation approaches already leads to good outcomes, there is a less clear role for this approach. Additionally, it is important to recognize limitations of the existing literature, which is often single-center, small cohort studies with significant patient selection bias and non-standardized approaches. Global initiatives such as the International Microtransplant Interest Group (IMIG) and others will help to standardize approaches and streamline workstreams to provide larger, multi-center studies and wider enrolment.

## Discussion

Notwithstanding that the exact mechanism of efficacy is not established, there is clearly a body of clinical literature spanning decades that third-party leukocyte exposure can improve leukemic outcomes, including after transplant, and that such leukocyte exposure can induce antigen-specific immune responses. It is our hypothesis that such responses might be manipulated to make this antigen-response tumor specific. This might make transplant better as the GVL is better focused to the leukemia and less toxic, as the antigen is tumor-specific and not an antigen also expressed on host tissues. This is the holy grail of transplant, to separate GVL from GVHD.

Adoptive T cell therapies including CAR and TCT approaches have shown us that antigen specific immune responses can control tumor and yet these therapies are limited by cost, by that the genetically manipulated cells are skewed to exhaustion and by that the cells are autologous and inhibited by the tumor microenvironment and by regulatory T cells that protect any tumor from the immune system. Using leukocytes to generate an antigen-specific immune response might overcome these limitations in both a cost effective and HLA-unrestricted way.

Directing T cells in this fashion requires that a T cell replete donor cell source is used during transplant and is not inhibited by intensified immune suppression after transplant, including post-transplant cyclophosphamide (PTCy) which has been used increasingly over recent years (45). Cord blood remains therefore an attractive cell source as it is performed T-cell replete and there is less chronic GVHD than with other approaches. Directing the leukemic response to disease-specific antigens would allow for selection of better matched cords since an allogeneic immune response is not required and therefore transplant becomes safer. We believe that the use of adjuvant non-engrafting leukocytes in the peri-transplant period is worthy of further evaluation, both in the pre-clinical setting to elucidate the exact mechanisms at play and in well designed and standardized multi-center clinical trials to provide an alternative and potentially hugely beneficial approach in treating those patients with highest-risk disease.

## Data Availability

The original contributions presented in the study are included in the article/supplementary material. Further inquiries can be directed to the corresponding author.
